# The complete chloroplast genome of *Ranunculus yunnanensis* (Ranunculaceae)

**DOI:** 10.1080/23802359.2021.2002211

**Published:** 2021-12-16

**Authors:** Rui Rao, Zhe-Fei Zeng, Yu Du, Chun-Min Mao, Lei Xie, Xue-Lian Guo, Liang-Liang Yue

**Affiliations:** a Yunnan Key Laboratory of Plateau Wetland Conservation, Restoration and Ecological Services, Southwest Forestry University, Kunming, PR China; b National Plateau Wetland Research Center, Kunming, PR China; c Technology Center of Kunming Customs, Kunming, PR China; d College of Nature Conservation, Beijing Forestry University, Beijing, PR China

**Keywords:** *Ranunculus yunnanensis*, chloroplast genome, phylogenomic

## Abstract

*Ranunculus yunnanensis* Franch is endemic in Yunnan and Sichuan Provinces, southwestern China. Here, we report the complete chloroplast (cp) genome of *R. yunnanensis.* The chloroplast genome is 156,050 bp in length, with 111 encoded genes, including 78 protein-coding genes, 29 *tRNA* genes, and four *rRNA* genes. Maximum-likelihood phylogenetic reconstruction using the existing data of *Ranunculus* shows that *R. yunnanensis* is revealed at the basal position of the marsh buttercup clade. This result has improved a better understanding of the internal relationship of the *Ranunculus*.

*Ranunculus yunnanensis* Franch is a perennial herb of *Ranunculus* only distributed in north, northwest Yunnan, and southwest Sichuan (Editorial Committee of Flora of China, Chinese Academy of Sciences 1980). This species is usually found in wet and marsh areas, producing typical buttercup flowers but longed *Halerpestes* like leaves. Although taxonomist has published many researches focusing on taxonomy and phylogeny of this genus, the classification, phylogeny, and evolutionary pattern of this large genus is still controversial due to its morphological plasticity, crossbreeding, polyploidy, and lack of molecular systematics (Hörandl et al. 2005; Emadzade et al. 2010; Li et al. 2019). The chloroplast (cp) genome is widely used to reconstruct phylogenetic relationships among plant groups because of its circle like linear pattern of genes, unique gene content and sequence conservation, and a large number of single-copy genes (Soltis and Soltis 1998; Gitzendanner et al. 2018; Yang et al. 2021). Till now, there are only 8 cp genomes have been published within this species abundant genus (Raubeson et al. 2007; He et al. 2019; Li et al. 2019). We need more information on molecular data to make the evolution of this genus more clear. In this study, we reported the complete cp genome sequence of *R. yunnanensis*.

Fresh leaves of the *R. yunnanensis* were collected from Shangri-La, Yunnan Province (113°24′06″ E, 23°43′04″ N). The voucher specimen (20190716-24) is deposited in the Museum of Beijing Forestry University (http://bjfc.bjfu.edu.cn/index.htm, Lei Xie, email: xielei_si@126.com). A sequencing library was sequenced using Illumina nova-seq 6000 platform. The cp genome was assembled from the de novo datasets using the GetOrganelle pipeline (Jin et al. 2020), using *R. reptans* (GenBank accession number: NC_036977.1) as the reference sequence. The cp genome of *R. yunnanensis* was annotated with Plastid Genome Annotator (PGA) (Qu et al. 2019). The annotated results were manually checked for the start and stop codons, and intron/exon boundaries of protein-coding genes using the Geneious Prime (Kearse et al. 2012). The circle molecule was converted into feature table files by GB2squine (Lehwark and Greiner 2019) and submitted to GenBank.

The complete cp genome of *R. yunnanensis* is a 156,050 bp cyclic molecule, composed of four different regions: a large single copy (LSC, 85,556 bp) region and a small single copy (SSC, 19,772 bp) region are separated by two reverse repeats (IR, 25,361 bp) regions. The genome contains 111 encoded genes, including 78 protein-coding genes, 29 *tRNA* genes, and four *rRNA* genes. The total GC content was 37.9%, while the GC contents in LSC, SSC, and IR regions were 36.1%, 31.5%, and 43.5%, respectively. Annotated cp genome sequence was submitted to GenBank with an accession number MZ703201.

Phylogenetic analysis was carried out in the software toolkit phylosuite (Zhang et al. 2020). All sequences were aligned using MAFFT version 7 (Katoh and Standley 2013). The best nucleotide substitution model was TIM + F+R3, revealed by ModelFinder (Kalyaanamoorthy et al. 2017). The IQ-TREE version 1.6.12 (Nguyen et al. 2015) was used for maximum likelihood (ML) reconstruction based on the selected model with a statistic of 5000 ultrafast bootstrap replications. The ML tree based on the existing data of *Ranunculus* shows that *R. yunnanensis* is at the basal position within the marsh buttercup species (Figure 1). Our research results further provide basic information for the phylogeny and biogeography of *Ranunculus*.

**Figure 1.**
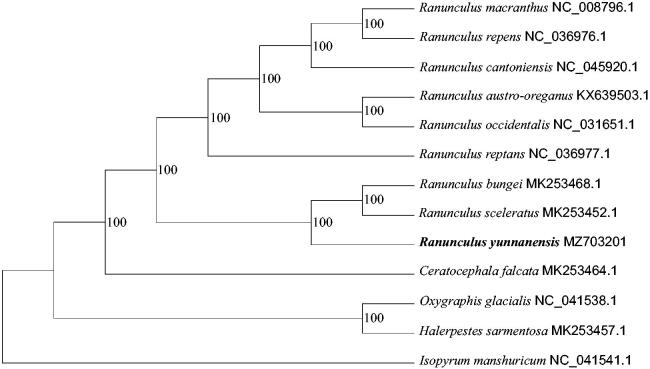
Maximum likelihood (ML) phylogenetic tree based on 13 complete cp genomes. The tree was rooted using *Isopyrum manshuricum*, Ranunculaceae, as outgroup. The bootstrap support values were marked above the branches.

## Data Availability

The genomic sequence data supporting the results of this study can be obtained in GenBank of NCBI at (https://www.ncbi.nlm.nih.gov) under the accession number MZ703201. The associated BioProject, SRA, and Bio-Sample numbers are PRJNA754365, SRR15497092, and SAMN20769473, respectively.
